# Retrospective observational study of salvage line ramucirumab monotherapy for patients with advanced gastric cancer

**DOI:** 10.1186/s12885-020-06865-7

**Published:** 2020-04-21

**Authors:** Sadayuki Kawai, Naoki Fukuda, Shun Yamamoto, Seiichiro Mitani, Katsuhiro Omae, Takeru Wakatsuki, Ken Kato, Shigenori Kadowaki, Daisuke Takahari, Narikazu Boku, Kei Muro, Nozomu Machida

**Affiliations:** 1 Division of Gastrointestinal Oncology, Shizuoka Cancer Center, 1007 Shimonagakubo, Nagaizumi, Sunto-gun, Shizuoka, 411-8777 Japan; 2 Department of Gastroenterology, Cancer Institute Hospital of Japanese Foundation For Cancer Research, 3-8-31, Ariake, Koto, Tokyo, 135-8550 Japan; 3 Division of Gastrointestinal Medical Oncology, National Cancer Center Hospital, 5-1-1 Tsukiji, Chuo-ku, Tokyo, 104-0045 Japan; 4 Department of Clinical Oncology, Aichi Cancer Center Hospital, 1-1 Kanokoden, Chikusa-ku, Nagoya, 464-8681 Japan; 5 Clinical Research Center, Shizuoka Cancer Center, 1007 Shimonagakubo, Nagaizumi, Sunto-gun, Shizuoka, 411-8777 Japan

**Keywords:** Stomach neoplasms, Drug therapy, Salvage therapy, Ramucirumab

## Abstract

**Background:**

Ramucirumab monotherapy as a second-line treatment for advanced gastric cancer (AGC) prolongs survival compared to the best supportive care. However, in clinical practice, ramucirumab monotherapy is sometimes used as third- or later-line treatment for AGC refractory to fluoropyrimidine and taxanes. This study evaluated the efficacy and safety of salvage-line ramucirumab monotherapy for treating AGC.

**Methods:**

The subjects of this retrospective study were advanced gastric or gastro-esophageal junction adenocarcinoma patients who received ramucirumab monotherapy after failure of 2 or more prior regimens containing fluoropyrimidine and taxanes but not ramucirumab.

**Results:**

From June 2015 to April 2017, 51 patients were enrolled. The median progression-free survival (PFS) and overall survival (OS) were 1.8 (95% confidence interval [CI] = 1.6–2.2) and 5.1 (95% CI = 4.0–6.8) months, respectively. The objective response and disease control rates were 2 and 17%, respectively. Grade 3 adverse events (AEs; e.g., anemia, fatigue, hypertension, proteinuria, intestinal bleeding) occurred in seven (13%) patients, but no grade 4 AEs and treatment-related deaths were observed. A neutrophil–lymphocyte ratio (NLR) of < 2.5 and previous gastrectomy were associated with better PFS.

**Conclusions:**

Salvage-line ramucirumab monotherapy has acceptable toxicity and comparable efficacy to second-line treatment; therefore, we consider physicians might choose this therapy as a salvage-line treatment option for AGC refractory to the standard therapies.

## Background

Gastric cancer is the third leading cause of death due to cancer worldwide [1]. Chemotherapy is the standard treatment for patients with advanced (unresectable or recurrent) gastric cancer (AGC). Although recent developments in chemotherapies for AGC have prolonged patients’ survival, their prognoses remain poor. The commonly used regimen for first-line treatment of AGC is combination chemotherapy, including fluoropyrimidine and platinum, which achieves a median progression-free survival (PFS) of ~ 6 months and a median overall survival (OS) of ~ 1 year [2–4].

In a randomized phase III “Ramucirumab Monotherapy for Previously Treated Advanced Gastric or Gastroesophageal Junction Adenocarcinoma” (REGARD) trial, administering ramucirumab, an anti–vascular endothelial growth factor receptor (anti-VEGFR) monoclonal antibody, as a second-line treatment prolonged OS compared to the placebo (hazard ratio [HR] = 0.774; 95% confidence interval (CI) = 0.605–0.991) [5]. In addition, in a “Ramucirumab plus Paclitaxel versus Placebo plus Paclitaxel for Previously Treated Advanced Gastric or Gastroesophageal Junction Adenocarcinoma” (RAINBOW) trial, combination chemotherapy, including ramucirumab and paclitaxel as a second-line treatment, prolonged OS compared to the placebo and paclitaxel regimen (HR = 0.807; 95% CI = 0.678–0.962) [6]. Therefore, ramucirumab and paclitaxel doublet therapy is currently considered a standard second-line treatment, and ramucirumab monotherapy is also considered a second-line treatment option for AGC.

In clinical practice, some patients with AGC refractory to taxanes (e.g., patients who had an early relapse or progression during or soon after taxane-containing perioperative treatment) underwent ramucirumab monotherapy as third- or later-line treatment. Recent evidence has demonstrated that a taxane-containing regimen is beneficial in a perioperative setting. Al-Batran et al. [7] showed that a combination regimen, including fluorouracil, leucovorin, oxaliplatin, and docetaxel (FLOT), for resectable gastric cancer patients in a perioperative setting prolongs OS compared to a combination regimen including epirubicin, platinum, and fluorouracil. Yoshida et al. [8] reported that S-1 and docetaxel as adjuvant therapy prolonged relapse-free survival compared to S-1 alone. For patients who have an early relapse, despite undergoing docetaxel-containing perioperative chemotherapy, second-line chemotherapy without taxane (e.g., irinotecan or nivolumab) is considered optimal because of cross-resistance between docetaxel and paclitaxel [9, 10]. In such situations, ramucirumab monotherapy would be administered as third- or later-line treatment.

There are few reports on the efficacy and safety of ramucirumab monotherapy as third- or later-line treatment. Therefore, this retrospective, observational study evaluated the efficacy and safety of ramucirumab monotherapy as third- or later-line treatment for AGC refractory or intolerant to fluoropyrimidines and taxanes.

## Methods

### Patients

This study is a multicenter retrospective observational study. AGC patients who underwent ramucirumab monotherapy as third- or later-line treatment between June 2015 and April 2017 at four institutions in Japan were enrolled in this study. All data were retrospectively collected from patients’ medical records. The inclusion criteria were as follows: age ≥ 18 years, histologically proven adenocarcinoma of the stomach or the gastroesophageal junction (GEJ), Eastern Cooperative Oncology Group (ECOG) performance status (PS) ≤ 2, and disease refractory to, or intolerant of, fluoropyrimidine and taxanes. The human epidermal growth factor receptor 2 (HER2) expression status was not considered. The exclusion criteria were as follows: severe bleeding, cardiovascular events, and uncontrolled comorbidities (e.g., hypertension, heart failure, or infection). Therefore, 51 patients were enrolled.

The study was approved by the ethics committees of all participating institutions based on biomedical research guidelines specified in the Declaration of Helsinki. Since the study was retrospective, informed consent was waived. Authors uploaded the information about this study and patients’ rights to the participating institutions’ websites.

### Treatment

All 51 patients underwent ramucirumab monotherapy at a dose of 8 mg/kg biweekly. Treatment was stopped in cases of disease progression, unacceptable toxicity, or patient refusal. The dose was reduced or treatment delayed at each physician’s discretion based on a specific patient’s condition and the severity of toxicity. The relative dose intensity (RDI) was calculated as follows:

$$ \mathrm{RDI}=\left(\mathrm{Total}\ \mathrm{actually}\ \mathrm{administered}\ \mathrm{dose}/\mathrm{Planned}\ \mathrm{dose}\ \mathrm{during}\ \mathrm{treatment}\right)\times 100 $$

### Evaluation and statistical analysis

The following data was collected from each patient: age, sex, ECOG PS, primary tumor location, tumor histology, HER2 status, metastatic sites, prior treatment, time that first-line treatment began, comorbidity, concomitant medication, and laboratory data at the start of ramucirumab monotherapy. The neutrophil–lymphocyte ratio (NLR) was calculated as follows:

$$ \mathrm{NLR}=\mathrm{Absolute}\ \mathrm{neutrophil}\ \mathrm{count}/\mathrm{Absolute}\ \mathrm{lymphocyte}\ \mathrm{count} $$

The platelet–lymphocyte ratio (PLR) was defined as follows:

$$ \mathrm{PLR}=\mathrm{Absolute}\ \mathrm{platelet}\ \mathrm{count}/\mathrm{Absolute}\ \mathrm{lymphocyte}\ \mathrm{count} $$

The cutoff values for the NLR and PLR were determined as 2.5 and 250, respectively, as described previously [11–13].

OS was calculated from the date of starting ramucirumab monotherapy until death due to any cause and censored at the last contact with surviving patients. PFS was calculated from the date of starting ramucirumab monotherapy until disease progression based on radiological images or clinical judgment and censored at the last contact with surviving patients without disease progression. Tumor responses were evaluated in patients with measurable lesions according to the Response Evaluation Criteria in Solid Tumors (RECIST) ver. 1.1 [14]. Toxicity was assessed according to the Common Terminology Criteria for Adverse Events (CTCAE) ver. 4.0. The PFS and OS were estimated using the Kaplan–Meier method.

The association between each patient’s background factors with the PFS or OS was estimated by univariate analysis using the log-rank test, and statistically significant background factors in univariate analysis were included in multivariate analysis using the Cox regression model. For categorical data, we again performed univariate analysis using Fisher’s exact test and multivariate analysis using the logistic regression model. Because our study was a retrospective exploratory analysis, sample size was not estimated a priori. All statistical analyses were conducted using EZR statistical software ver. 1.30 [15], and all statistical tests were two sided. *P* < 0.05 was considered statistically significant.

## Results

### Patients’ characteristics

Table 1 shows the backgrounds of the 51 patients enrolled in this study. Briefly, the patients’ median age was 67 years (range = 39–84 years), and 41 (80%) patients were male. Of the 51 patients, 43 (84%) had ECOG PS = 0 or 1. 35 (69%) were histologically diagnosed with intestinal-type adenocarcinoma, 17 (33%) were HER2 positive, and 29 (57%) had ≥2 metastatic sites.

**Table 1:** Patients’ characteristics in this study (*n* = 51)

		Number	%
Age	Median (range)	67 (39–84)	
Sex	Male	41	80
	Female	10	20
ECOG PS	0	4	8
	1	39	76
	2	8	15
Location of primary tumor	Stomach	43	84
	GEJ	8	16
Histology	intestinal	35	69
	diffuse	16	31
HER2 overexpression	(+)	17	33
Previous gastrectomy	(+)	24	47
Number of metastatic sites	0–1	22	43
	2 or more	29	57
Metastatic sites	Lymph nodes	9	18
	Liver	33	65
	Lung	11	22
	Peritoneum	23	45
Ascites	(+)	21	41
Time from initiation of 1st line therapy	≥ 2 years	22	43
Number of prior therapies	2	7	14
	3	25	49
	4	12	24
	5	7	14
Agents of prior therapies	S-1	43	84
	Capecitabine	14	27
	5-FU	6	12
	Cisplatin	39	76
	Oxaliplatin	17	33
	Paclitaxel	46	90
	Docetaxel	9	18
	Irinotecan	41	80
	Trastuzumab	15	29
	ICI	11	21
	Others	12	23
Concomitant use of antiplatelet or anticoagulation therapy	(+)	5	10
Concomitant use of NSAIDs	(+)	11	22
History of hypertension	(+)	16	31
Neutrophil-lymphocyte ratio	< 2.5	16	31
Platelet-lymphocyte ratio	< 250	29	57
ALP	< WNL	22	43
CRP	< WNL	15	29
LDH	< WNL	19	57

*Abbreviations*: *ECOG PS* Eastern Cooperative Oncology Group performance status, *HER2* human epidermal growth factor receptor 2, *GEJ* gastroesophageal junction, *ICI* immune checkpoint inhibitor, *NSAIDs* nonsteroidal anti-inflammatory drugs, *ALP* alkaline phosphatase, *CRP* C-reactive protein, *LDH* lactate dehydrogenase, *WNL* within normal limits

Forty-four (86%) patients underwent ≥3 prior chemotherapies, 44 (86%) received S-1 as fluoropyrimidine, 39 (76%) were administered cisplatin as platinum in first-line treatment, 46 (90%) were administered paclitaxel as taxane in second-line treatment, and 11 (21%) were administered immune checkpoint inhibitors (ICIs). The median time from starting first-line treatment was 22.2 months (range = 8.4–52.1 months). In addition, 5 (10%) patients were administered antiplatelet or anticoagulant drugs because of a past history of ischemic heart disease or stroke, 11 (21%) were administered nonsteroidal anti-inflammatory drugs (NSAIDs) for cancer pain, and 16 (31%) had a history of hypertension.

### Treatments

The median number of ramucirumab monotherapy administrations in each patient was 4 cycles (range = 1–31 cycles), with a total of 281 cycles in all 51 patients. No patient required a dose reduction in subsequent courses. However, administration of ramucirumab monotherapy was delayed in 12 (23%) patients (total 18 cycles) because of patients’ wishes, a holiday, or minor adverse events (AEs) such as grade 2 hypertension, grade 2 proteinuria, and grade 1 fever. The median RDI of ramucirumab monotherapy in all patients was 100% (range = 76–100%). Of the 51 patients, ramucirumab monotherapy was discontinued in 47 (92%) patients because of disease progression (44 patients, 86%) and AEs (3 patients, 6%; grade 3 small intestinal hemorrhage in 1 patient and grade 3 proteinuria in 2 patients). As subsequent therapy, best supportive care was performed in 29 (57%) patients, and chemotherapies were administered to 18 (35%) patients, including a fluoropyrimidine rechallenge in 7 (14%), irinotecan in 5 (10%), and ICIs in 3 (6%) patients.

### Efficacy

**Fig. 1:**
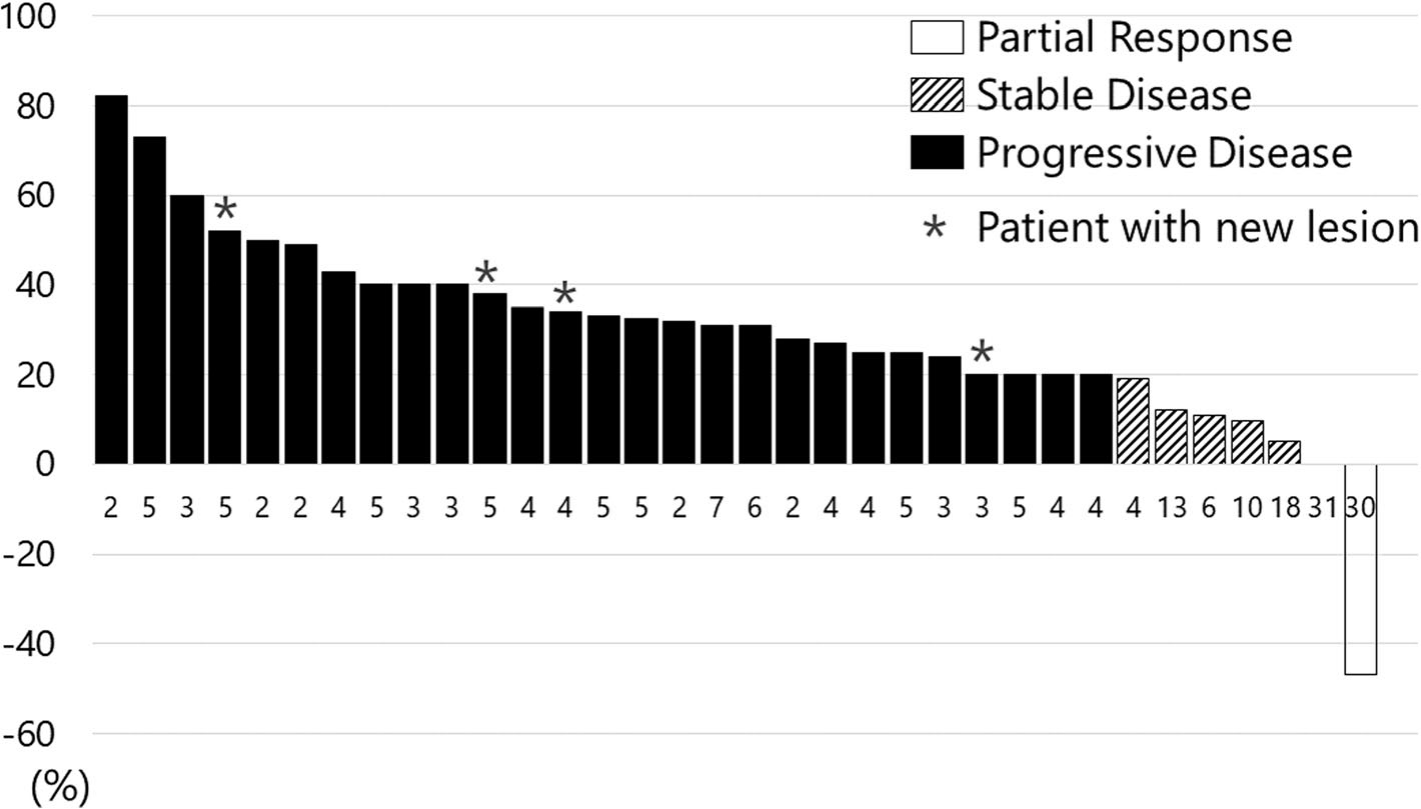
Waterfall plot of tumor response for evaluable patients (*n* = 34). The numbers beside each bar are ramucirumab administration cycles. *Patients with a new lesion at the first evaluation

**Fig. 2:**
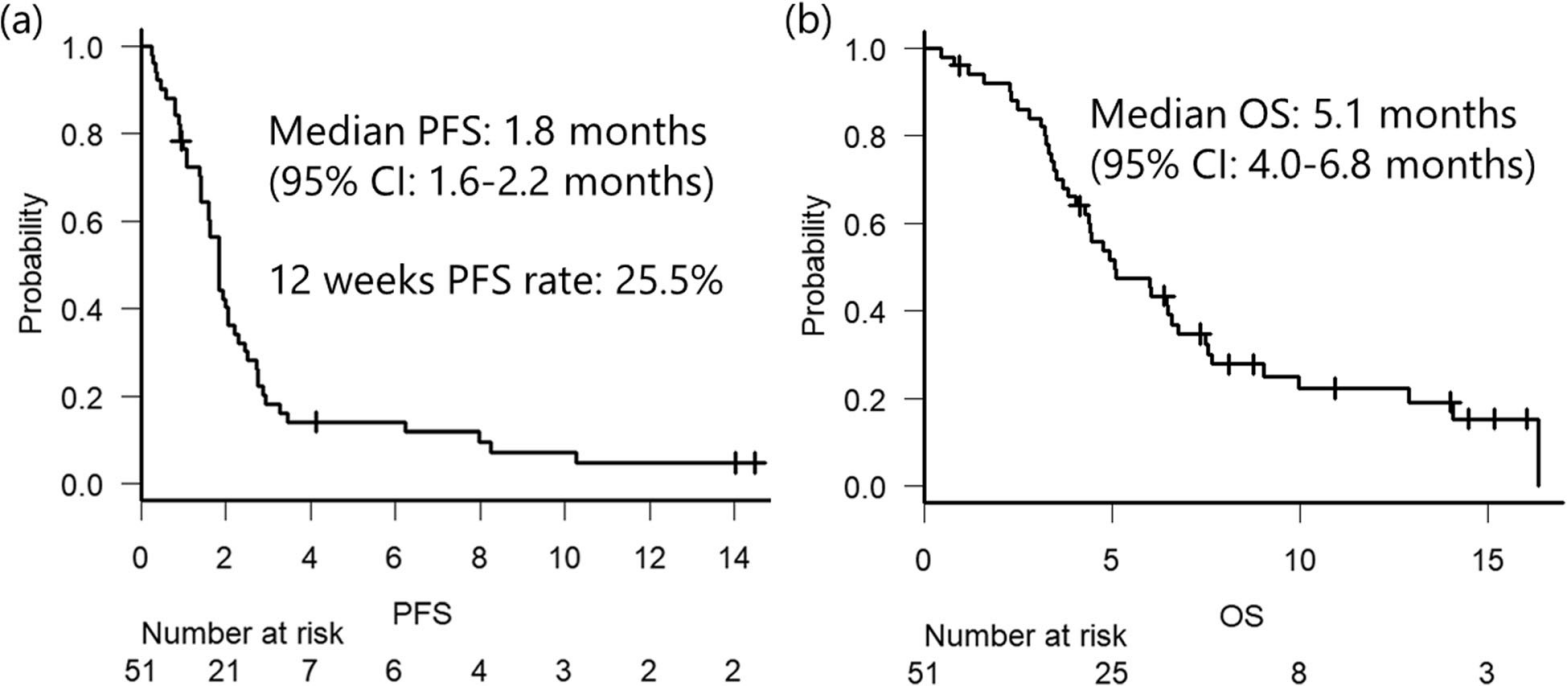
Kaplan–Meier curves of PFS (**a**) and OS (**b**). PFS, progression-free survival; OS, overall survival

Of the 42 (82%) patients with measurable lesions, we were unable to evaluate the tumor response in 8 (16%) patients because of disease progression, clinically judged, in 5 patients, discontinuation due to AEs in 2, and treatment before evaluation by imaging in 1. In addition, 1 patient achieved partial response, while 6 patients showed stable disease, resulting in a response rate (RR) of 2% and a disease control rate (DCR) of 17%. For proportions of change in target lesions at the best response, compared to the baseline, please refer to the waterfall plot in Fig. 1. After a median follow-up period of 8.9 months, the median PFS was 1.8 months (95% CI = 1.6–2.2) and the median OS was 5.1 months (95% CI = 4.0–6.8) (Fig. 2).

### Prognostic factors

Patients with NLR <  2.5 and those with previous gastrectomy had longer PFS (Table 2). Although no background factors were identified as significant prognostic factors for OS (Table 3), NLR <  2.5 was associated with longer OS. Patients with NLR < 2.5 also tended to have a high DCR (4 of 11, 36%) compared to NLR ≥ 2.5 (3 of 23, 13%). We also investigated the relationship between prior therapies and the efficacy of ramucirumab. However, multivariate analysis did not showed significant difference between them (data not shown).

**Table 2:** Exploratory analysis of prognostic factors for PFS

Covariates		Univariate analysis		Multivariate analysis	
		HR (95% CI)	***P***	HR (95% CI)	***P***
Number of prior therapies	≥4	1.37 (0.73–2.56)	0.324		
Sex	male	0.65 (0.31–1.37)	0.261		
Age	> 66	0.97 (0.53–1.76)	0.931		
ECOG PS	2	1.57 (0.72–3.41)	0.251		
Location of primary tumor	Stomach	0.59 (0.35–1.37)	0.222		
Histology	diffuse	0.85 (0.44–1.63)	0.636		
HER2 overexpression	(+)	1.52 (0.80–2.87)	0.192		
Concomitant use of NSAIDs / antiplatelets / anticoagulation therapy	(+)	1.12 (0.60–2.11)	0.709		
History of hypertension	(+)	0.53 (0.27–1.04)	0.066		
Number of metastatic sites	≥2	1.51 (0.83–2.75)	0.17		
Ascites	(+)	1.78 (0.97–3.26)	0.061		
Peritoneal metastasis	(+)	1.32 (0.74–2.37)	0.339		
Previous gastrectomy	(+)	0.48 (0.26–0.89)	0.019	0.50 (0.27–0.94)	0.031
Time from initiation of 1st line therapy	≥2 years	0.53 (0.29–0.98)	0.043	0.66 (0.35–1.25)	0.208
Prior history of ICI	(+)	1.03 (0.52–2.06)	0.92		
ALP	≥WNL	1.08 (0.59–1.98)	0.797		
CRP	≥WNL	1.34 (0.71–2.51)	0.359		
LDH	≥WNL	1.42 (0.74–2.74)	0.285		
NLR	< 2.5	0.35 (0.16–0.74)	0.002	0.38 (0.17–0.87)	0.017
PLR	< 250	0.58 (0.32–0.97)	0.033	0.83 (0.44–1.56)	0.413

*Abbreviations*: *PFS* progression-free survival, *HR* hazard ratio, *CI* confidence interval, *ECOG PS* Eastern Cooperative Oncology Group performance status, *HER2* human epidermal growth factor receptor 2, *NSAIDs* nonsteroidal anti-inflammatory drugs, *ICI* immune checkpoint inhibitor, *ALP* alkaline phosphatase, *CRP* C-reactive protein, *LDH* lactate dehydrogenase, *WNL* within normal limits

**Table 3:** Exploratory analysis of prognostic factors for OS

Covariates		Univariate analysis		Multivariate analysis	
		HR (95% CI)	***P***	HR (95% CI)	***P***
Number of prior therapies	≥4	1.56 (0.81–2.99)	0.176		
Sex	Male	0.66 (0.30–1.45)	0.304		
age	> 66	0.87 (0.46–1.64)	0.671		
ECOG PS	2	1.61 (0.71–3.68)	0.252		
Location of primary tumor	Stomach	1.12 (0.46–2.70)	0.795		
Histology	diffuse	1.09 (0.55–2.16)	0.801		
HER2 overexpression	(+)	1.59 (0.81–3.12)	0.171		
Concomitant use of NSAIDs/antiplatelets/anticoagulation therapy	(+)	1.60 (0.80–3.18)	0.18		
History of hypertension	(+)	0.71 (0.35–1.47)	0.371		
Number of metastatic sites	≥2	1.86 (0.97–3.59)	0.061		
Ascites	(+)	1.88 (0.99–3.56)	0.05		
Peritoneal metastasis	(+)	0.79 (0.41–1.49)	0.468		
Previous gastrectomy	(+)	0.71 (0.37–1.35)	0.297		
Time from initiation of 1st line therapy	≥2 years	0.67 (0.35–1.31)	0.249		
Prior history of ICI	(+)	1.45 (0.68–3.10)	0.329		
ALP	≥WNL	1.28 (0.67–2.46)	0.444		
CRP	≥WNL	2.18 (1.02–4.61)	0.041	1.72 (0.76–3.89)	0.187
LDH	≥WNL	2.02 (1.04–3.91)	0.037	1.66 (0.74–3.70)	0.149
NLR	< 2.5	0.44 (0.20–0.93)	0.033	0.56 (0.28–1.12)	0.089
PLR	< 250	0.53 (0.28–1.01)	0.052		

*Abbreviations*: *OS* overall survival, *HR* hazard ratio, *CI* confidence interval, *ECOG PS* Eastern Cooperative Oncology Group performance status, *HER2* human epidermal growth factor receptor 2, *NSAIDs* nonsteroidal anti-inflammatory drugs, *ICI* immune checkpoint inhibitor, *ALP* alkaline phosphatase, *CRP* C-reactive protein, *LDH* lactate dehydrogenase, *WNL* within normal limits

### Safety

Table 4 lists the hematological and non-hematological AEs associated with ramucirumab monotherapy. Overall, 38 of 51 (74%) patients had at least one treatment-related AE, while 7 (13%) had grade 3 AEs, including anemia (2 patients, 4%), fatigue (1 patient, 2%), hypertension (2 patients, 4%), proteinuria (2 patients, 4%), and bleeding (1 patient, 2%). We did not observe grade 4 AEs and treatment-related death. Univariate analysis showed no significant relationship between each AE and the patient’s background (data not shown).

**Table 4:** Hematological and non-hematological AEs associated with ramucirumab treatment

	Any grade (%)	Grade ≥ 3 (%)
Any adverse events	38 (74%)	7 (13%)
Leukopenia	4 (8%)	0
Neutropenia	9 (18%)	0
Anemia	18 (35%)	2 (4%)
Thrombocytopenia	4 (8%)	0
Fatigue	19 (37%)	1 (2%)
Anorexia	19 (37%)	1 (2%)
Hyponatremia	1 (2%)	1 (2%)
Hypertension	18 (35%)	2 (4%)
Proteinuria	15 (29%)	2 (4%)
Thrombocytopenic events	1 (2%)	0
Bleeding	5 (10%)	1 (2%)
Gastrointestinal perforation	0	0

*Abbreviations*: *AE* adverse event

## Discussion

As second-line treatment for AGC, the REGARD trial showed a median PFS of 2.1 months and a median OS of 5.2 months [5]. A Japanese trial on ramucirumab monotherapy as second-line treatment showed a median PFS of 1.6 months and a median OS of 7.3 months [16]. This favorable OS, despite a PFS similar to that in this study, might be obtained by patient selection and subsequent treatments. We believe that the efficacy of ramucirumab monotherapy as third- or later-line treatment, as shown in this study, is comparable to previous studies that reported ramucirumab monotherapy as second-line treatment.

Paulson et al. [17] reported retrospective observational data on ramucirumab monotherapy for AGC patients in the United States. They studied 115 patients who underwent ramucirumab monotherapy, 38.3% of whom underwent ramucirumab monotherapy as third- or later-line treatment. The authors reported that the median duration of therapy was 1.87 months; the median OS and RR were not reported. Murahashi et al. [18] retrospectively analyzed the efficacy and safety of ramucirumab monotherapy as second- or later-line treatment at a single institution. The authors reported that 15 of their 19 patients underwent ramucirumab monotherapy as third- or later-line treatment. Overall, the median OS, median PFS, RR, and grade 3 AE incidence were 12.9 months, 2.1 months, 7.7, and 10%, respectively. Our results were comparable to these reports, suggesting prolonged survival after salvage-line ramucirumab monotherapy for AGC.

The NLR is reportedly a poor prognostic factor in several cancers, such as colon, breast, pancreatic, and gastric cancer [19–22], in a metastatic or an adjuvant setting. The NLR is an inflammation marker; a high NLR reflects enhanced systemic inflammation and is associated with reduced tumor-specific immunity, such as decreased tumor-infiltrating lymphocytes in a tumor [23]. Neutrophilia inhibits the cytolytic activity of immune cells [24] and increases the production of factors that promote tumor growth, such as VEGF and hepatocyte growth factor [25]. Lymphocytopenia is often observed in patients with advanced disease, indicating a state of immunosuppression [26]. From a pooled analysis of REGARD and RAINBOW trials, Fuchs et al. [27] reported that 12 clinical and laboratory factors, including a high neutrophil and low lymphocyte count, at the start of ramucirumab monotherapy are negative prognostic factors for OS. From Korean retrospective cohort data of ramucirumab mono- or combination therapy, Jung et al. [28] reported that the NLR and albumin are independent prognostic factors for the PFS and OS. Our results also supported these findings.

Although studies have evaluated several predictive biomarkers of antiangiogenic therapy, no reproducible biomarkers, including soluble VEGF-C, VEGF-D, VEGFR1, and VEGFR3, have been identified in the REGARD trial [29]. Some studies have suggested the NLR’s predictive value. Cho et al. [30] reported that AGC patients with low NLRs have higher DCRs for cytotoxic chemotherapy compared to patients with high NLRs. Similarly, the association between the NLR and the tumor response of chemotherapy has been reported in several neoplasms, such as colon, head and neck, and lung cancer [20, 31, 32]. The immunological response caused by chemotherapy could be a reason for these findings. Compared to patients with high NLRs, patients with low NLRs are likely to have the inflammatory reaction environment mediated by lymphocytes [30]. Ogata et al. [33] and Bagley et al. [34] reported that the NLR might predict the tumor response of nivolumab in gastric cancer and non–small cell lung cancer, respectively. Tada et al. [35] reported that ramucirumab suppresses tumor infiltration of effector regulatory T-cells (eTreg) and decreases programmed cell death protein 1 (PD-1) expression by CD8+ T-cells, subsequently enhancing antitumor immunity. Therefore, the NLR might also predict the ramucirumab-induced immunological antitumor effect.

In third-line treatment, the patients’ performance status is generally worse than second-line treatment. In the REGARD and Japanese prospective phase II trials, 28 and 66% of patients had ECOG PS = 0, respectively [5, 16], compared to 8% in our study; in addition, 15% of the patients had ECOG PS = 2. In the REGARD and Japanese prospective phase II prospective trials, all AEs (any grade) occurred in 94 and 97% of patients (74% in our study), respectively, while AEs of ≥ grade 3 occurred in 57 and 25% of patients (13% in our study), respectively. These findings suggest that ramucirumab monotherapy is also feasible as salvage-line treatment.

This study had a few limitations. It was retrospective in nature, had a small sample size, and had selection bias. The median duration from the start of first-line treatment to the start of ramucirumab monotherapy was 21.9 months, which was long compared to the average of OS of AGC patients, suggesting that we selected patients with slowly growing cancers or cancers highly responsive to prior chemotherapy. However, because we analyzed multicenter data, our findings seem generalized to patients in clinical practice. Secondly, we could not assess patient’s quality of life (QOL). QOL is one of the most important outcomes for the patients in salvage-line treatments. In phase III REGARD trial, ramucirumab monotherapy demonstrated the better QOL compared with best supportive care in second-line. In our study, toxicities of salvage-line ramucirumab were comparable to the second-line setting, and actually, 93% of patients could continue the treatment until disease progression. Therefore, we speculated salvage-line ramucirumab might have similar effect for QOL, however, further investigation is needed.

## Conclusions

Ramucirumab monotherapy demonstrates acceptable efficacy and feasibility as third- or later-line treatment for AGC. We consider physicians might choose this therapy as a salvage-line treatment option for AGC refractory to the standard therapies.

## Data Availability

The datasets used and/or analyzed during the current study are available from the corresponding author on reasonable request.

## Abbreviations

AGC: Advanced gastric cancer
PFS: Progression-free survival
OS: Overall survival
CI: Confidence interval
AE: Adverse event
NLR: Neutrophil–lymphocyte ratio
VEGF: Vascular endothelial growth factor
VEGFR: Vascular endothelial growth factor receptor
HR: Hazard ratio
GEJ: Gastroesophageal junction
ECOG: Eastern Cooperative Oncology Group
PS: Performance status
HER2: Human epidermal growth factor receptor 2
RDI: Relative dose intensity
PLR: Platelet–lymphocyte ratio
RECIST: Response Evaluation Criteria in Solid Tumors
CTCAE: Common Terminology Criteria for Adverse Events
NSAIDs: Nonsteroidal anti-inflammatory drugs
ICIs: Immune checkpoint inhibitors
RR: Response rate
DCR: Disease control rate
PD-1: Programmed cell death protein 1
QOL: Quality of life
ALP: Alkaline phosphatase
CRP: C-reactive protein
LDH: Lactate dehydrogenase
WNL: Within normal limits
